# In vivo effect of statins on the expression of the HIV co-receptors CCR5 and CXCR4

**DOI:** 10.1186/1742-6405-10-10

**Published:** 2013-05-01

**Authors:** Edwin A Higuita, Fabián A Jaimes, Maria T Rugeles, Carlos J Montoya

**Affiliations:** 1 Immunovirology Group, School of Medicine, University of Antioquia, Medellin, Colombia; 2 Department of Internal Medicine and Academic Group of Clinical Epidemiology (GRAEPIC), School of Medicine, University of Antioquia, Medellín, Colombia; 3 Clinical Research Unit, Hospital Pablo Tobón Uribe, Medellin, Colombia

**Keywords:** HIV infection, Statins, CCR5, CXCR4, Viral tropism, Chemokines

## Abstract

**Background:**

During the HIV-1 replication cycle, several molecules including chemokine receptors and cholesterol are crucial, and are therefore potential targets for therapeutic intervention. Indeed statins, compounds that inhibit cellular synthesis of cholesterol and have anti-inflammatory and immunomodulatory properties were shown to inhibit HIV-1 infection by R5 tropic strains but not by X4 strains in vitro, mainly by altering the chemokine receptor/ligands axes. Therefore, the objective of this study was to characterize in vivo, the capacity of statins to modulate in HIV seronegative and chronically HIV-1-infected adults the expression of CCR5 and CXCR4, of their ligands and the tropism of circulating HIV-1 strains.

**Methods:**

Samples from asymptomatic HIV-1-infected adults enrolled in a clinical trial aimed at evaluating the antiretroviral activity of lovastatin were used to evaluate in vivo the modulation by lovastatin of CCR5, CXCR4, their ligands, and the shift in plasma viral tropism over one year of intervention. In addition, ten HIV negative adults received a daily oral dose of 40 mg of lovastatin or 20 mg of atorvastatin; seven other HIV negative individuals who received no treatment were followed as controls. The frequency and phenotype of immune cells were determined by flow-cytometry; mRNA levels of chemokine receptors and their ligands were determined by real-time PCR. Viral tropism was determined by PCR and sequencing, applying the clonal and clinical model of analyses.

**Results:**

Our study shows that long-term administration of lovastatin in HIV-infected individuals does not induce a shift in viral tropism, or induce a significant modulation of CCR5 and CXCR4 on immune cells in HIV-infected patients. Similar results were found in HIV seronegative control subjects, treated with lovastatin or atorvastatin, but a significant increase in CCL3 and CCL4 transcription was observed in these individuals.

**Conclusions:**

These findings suggest that long-term administration of statins at therapeutic doses, does not significantly affect the expression of HIV-1 co-receptors or of their ligands. In addition it is important to point out that based on the results obtained, therapeutic administration of statins in HIV-infected patients with lipid disorders is safe in terms of selecting X4 strains.

## Background

Despite the fact that highly active antiretroviral therapy (HAART) is a clinically accepted and cost-effective intervention [1], its efficiency is hampered by limitations in therapy adherence and the emergence of viral resistance, facts closely related to adverse effects and toxicity [2,3]. Thus, it is necessary to develop new medications and therapeutic strategies, with novel mechanisms of action to overcome the limitations of HAART.

HIV infection involves an initial interaction between the viral envelope glycoprotein gp120 and the CD4 molecule on the surface of target cells, followed by a second interaction with a co-receptor; for HIV-1, the classical co-receptor function is provided by one of two different molecules, the C-C chemokine receptor 5 (CCR5) or the C-X-C chemokine receptor 4 (CXCR4), which have a normal physiological function serving as receptors for soluble chemokines [4,5]. The critical role of these molecules in HIV infection encouraged the design of anti-HIV drugs aimed at blocking this molecular interaction [6-8]. Some molecules with ability to exert a non-competitive, allosteric, inhibition of the CCR5 receptor have been identified; among them, maraviroc is the first CCR5 antagonist approved for clinical use in HIV-infected patients, and its efficiency has been established in clinical trials [9,10]. However, before receiving maraviroc, it is mandatory to first identify the co-receptor used by the strains infecting each patient, due to the risk on selecting the more pathogenic strains that use the CXCR4 co-receptor.

On the other hand, almost every aspect of the HIV-1 life cycle relies on cholesterol [11-16]; in the absence of this lipid, the attachment of HIV-1 to its host cells is severely impaired because the clustering of HIV-1 receptor/co-receptor in lipid rafts is hindered [17-19], and the conformational status and functions of CCR5 and CXCR4 are critically affected [18]. Indeed, in vitro studies have demonstrated that, in the absence of cholesterol, virus-cell fusion is greatly diminished, viral transcytosis is inhibited, production and budding of viral particles are reduced, and cell signaling is altered [12,20,21]. Cellular synthesis of cholesterol may be effectively inhibited by statins, medications that are competitive analogues of the substrate of the 3-hydroxy-3-methylglutaryl coenzyme A (HMG-CoA) reductase, an enzyme that catalyzes the conversion of hydroxyl-methyl-glutarate into mevalonic acid, a precursor for the biosynthesis of cholesterol and isoprenoids [22-24]. Moreover, a previous in vitro study demonstrated that CD4+ T-cell incubation with statins reduced the level of CCR5 expression in these cells isolated from HIV negative individuals, while it increased the secretion of its natural ligand, RANTES (CCL5), thereby inhibiting HIV-1 infection by R5 tropic strains but no by X4 strains [25]. Consequently, it is important to verify if this effect is reproducible in vivo, mainly because statins are safe medications with other anti-inflammatory and immunomodulatory properties, that could be of potential benefit in the treatment of HIV-1-infected individuals [26-30].

Nevertheless, a therapy with medications that inhibit the surface expression of CCR5 in the absence of complete control of HIV replication, could promote the positive selection of X4 strains of HIV, considered more pathogenic [31-33]. Thus, it is important to determine if the in vivo administration of statins promotes a change in viral tropism with a shift from R5 to X4 strains of HIV-1, as this might alter the natural course of HIV-1 infection and increase the risk of rapid progression to AIDS.

This study was aimed at characterizing the in vivo capacity of statins to modulate, in HIV seronegative and chronically HIV-1 infected adults, the following parameters: i) expression of the HIV-1 co-receptors CCR5 and CXCR4, ii) levels of their ligands RANTES (CCL5), MIP-1α (CCL3), MIP-1β (CCL4) and SDF-1 (CXCL12), and iii) tropism of circulating HIV-1 strains.

## Methods

### Subjects of study

Samples from chronic asymptomatic HIV-1-infected adults enrolled in a randomized clinical trial aimed at determining the antiretroviral and immunomodulatory activity of lovastatin (LIVE study, NCT00721305) [34], were used to evaluate in vivo the modulation by lovastatin of: i) CCR5 and CXCR4 expression on peripheral blood mononuclear cells (PBMC; lovastatin group n = 41; placebo group n = 43), and ii) the shift in plasma viral tropism throughout one year of intervention (lovastatin n = 44; placebo n = 47). These patients were HAART naïve, had a detectable viral load (>50 and <100.000 RNA copies/ml) and CD4+ T-cell counts higher than 350 cells/μl; the exclusion criteria were previously defined [35].

HIV negative adults, were invited to receive a daily supervised oral dose of lovastatin (40 mg/d, n = 10) or atorvastatin (20 mg/d, n = 9); seven other individuals received no intervention but had the same follow-up as the intervened HIV negative controls for the steady state variation in the expression of the HIV-1 co-receptors.

The study was conducted in accordance with the Declaration of Helsinki, and the protocol was approved by the Institutional Ethical Review Board of the University of Antioquia. All participants provided signed informed consent, prepared according to the Colombian legislation (Resolution 008430 of 1993), before participating in the study.

### Statins

Tablets of lovastatin (20 mg), atorvastatin (20 mg) and placebo were kindly provided by Laproff Laboratories (Sabaneta, Colombia). The daily dose of these interventions was administered after the last meal at night, for 45 days in HIV negative controls and 360 days in HIV-infected patients from the LIVE study. The biological activity, safety and tolerance of statins were controlled by clinical evaluation, pharmacotherapeutic follow-up by a pharmacist, and by periodic testing of liver enzymes, creatine phosphokinase (CPK) and creatinine, and by performing a serum lipid profile. The intervention was stopped in the presence of any serious adverse drug event, a three-fold increase in serum hepatic aminotransferases or a five-fold increase in CPK.

### Flow cytometry protocols

The frequency and surface phenotype of CD4+ T cells and CD3-/CD4+ monocytes in whole peripheral blood samples were determined by flow cytometry; fluorochrome-labeled mouse monoclonal antibodies (mAbs) against human molecules were from Becton Dickinson (BD, San Jose, CA): CD3 FITC (clone HIT3a), CD4 APC (clone RPA-T4), CD184 PE-Cy5 (clone 12G5), CD195 PE-Cy5 (clone 2D7/CCR5) and the corresponding isotype control antibodies. Briefly, 150 μl of blood were incubated with specific mAbs for 25 min at room temperature in the dark; the erythrocytes were then lysed by incubating for 10 min with 2 ml of 1X Facs lysing solution (Becton Dickinson). The cells were washed twice with 2 ml of cold phosphate-buffered saline (PBS) and fixed with 250 ml of 2% paraformaldehyde. All the preparations were stored at 4°C until acquisition in the cytometer FACS CANTO-II (Becton Dickinson).

Acquisition analyses were performed using the BD FACSDiva, version 6.1.2. Lymphocyte and monocyte gates, identified by side (SSC) versus forward (FSC) light scatter, were used to analyze cell populations identified as CD3^+^/CD4^+^ and CD3^-/^CD4^+^ cells, respectively; the surface expression of CCR5 and CXCR4 molecules was analyzed in each cell population; dead cells were excluded from the analysis through SSC vs. FSC.

### CD4+ T cell isolation and real time PCR

PBMCs were isolated from heparin anti-coagulated peripheral blood by centrifugation in a Ficoll-Hypaque (Sigma, MO) gradient; CD4+ T lymphocytes were obtained by negative selection using the CD4+ T cell isolation kit II (Miltenyi Biotech, Auburn, CA), according to manufacturer’s instructions. From these CD4+ T lymphocytes, total RNA was extracted using the Quiagen RNA tissue extraction kit (Quiagen, CA) following the manufacturer’s instructions. The amount and purity of the RNA were determined by spectrometry at 260/280 nm. Total RNA was treated with DNase-I (Fermentas, St. Leon-Rot, Germany), and the DNA copy was synthesized using the RevertAid™ First Strand cDNA Synthesis kit (Fermentas®, Thermo Fisher Scientific, MA), according to manufacturer’s instructions.

For real time PCR, each 20 μl of mixture consisted of 0.5 μl of cDNA, 10 μl SYBR® Green PCR Master Mix (Invitrogen, CA) and primers (0.4 μM each). The primer sequences for CCR5, CXCR4, CCL3 (MIP-1alpha), CCL4 (MIP-1beta), CCL5 (RANTES), and CXCL12 (SDF-1) were designed using the PrimerBlast Tool (NCBI web site http://www.ncbi.nlm.nih.gov/). The target for each of this set of primers is directed against exon junctions, to avoid amplifying unspliced mRNA and/or genomic DNA (Table 1); the same cycling profiles were used for all sets of primers according to the SYBR® Green PCR Master Mix instructions (50°C for 2 min, followed by 95°C for 10 min and 40 cycles of 95°C for 15 sec and 60°C for 1 min).

**Table 1:** Primer pair sequences to detect expression of CCR5, CXCR4, CCL3, CCL4, CCL5 and CXCL12

**Primer name**	**Target mRNA**	**Primer sequence 5’-3’**	**Product size (bp)**
CCR5F	Exon 2a CCR5	TGATTTGCACAGCTCATCTGGCCA	169
CCR5R	Exon 3 CCR5	CGGGCTGCGATTTGCTTCACATT	
CXCR4F	Exon 1 CXCR4	AGTGACGCCGAGGGCCTGAG	150
CXCR4R	Exon 2b CXCR4	ACGGAAACAGGGTTCCTTCATGGA	
CCL3F	Exon 1 CCL3	CTCTGCAACCAGTTCTCTGCATCA	151
CCL3R	Exon 2/3 junction CCL3	TGGTTAGGAAGATGACACCGGGCT	
CCL4F	Exon 1 CCL4	CTGCCTTCTGCTCTCCAGCG	134
CCL4R	Exon 2 CCL4	GGAGCAGAGGCTGCTGGTCT	
CCL5F	Exon 1 CCL5	CCATGAAGGTCTCCGCGGCA	126
CCL5R	Exon 2 CCL5	GTGGGCGGGCAATGTAGGCAA	
CXCL12F	Exon 2/3b junction CXCL12	CCCTTCAGATTGTAGCCCGGCTG	209
CXCL12R	Exon 3b CXCL12	CTCATGGTTAAGGCCCCCTCCCC	

Glyceraldehyde 3-phosphate dehydrogenase (GAPDH) amplification was used to normalize the RNA content of the corresponding transcripts analyzed, and the result was expressed as relative units of transcription (1.8^[∆Ct Target gene-∆Ct GAPDH]^). We included a melting curve to confirm the specificity of the PCR product. All real-time RT-PCR amplifications were performed using the CFX96 real-time system and data analysis using the software CFX Manager Version: 1.5.534.0511 (Bio-Rad, Hercules, CA).

### Determination of viral tropism

Predictions of co-receptor usage by HIV strains have shown comparable results when performed using either plasma virus or proviral sequences [36]. Genomic DNA was isolated from PBMCs at months 0 and 12 of lovastatin or placebo treatment; briefly, PBMCs were washed twice with PBS and incubated overnight with White Cells Lysis Buffer (Tris–HCl 0.02 M; EDTA 0.02 M; NaCl 0.02 M; SDS 0.2%), and the genomic DNA was isolated using the phenol–chloroform technique. The amount and purity of the DNA samples were determined by spectrometry at 260/280 nm.

For proviral HIV-1 amplification, a nested PCR technique was used as previously described [37]. In the first round, the reaction mixture of 25 μl included 0.5 μg of genomic DNA, 0.4 nM of primer ED3, 0.4 nM of primer ED14, and 12.5 μl of 2X PCR Master Mix (Fermentas® Thermo Fisher Scientific, MA). For the second round of PCR, the same proportion of reactants was used for a reaction of 50 μl including the primers ED31 and ED33. In both PCR rounds, the reaction was subjected to a prePCR: 94°C for 3 min, followed by 4 cycles at 94°C for 60 sec, and 55°C for 60 sec. Then, 32 PCR cycles at 94°C for 15 sec, 55°C for 45 sec and 72°C for 60 sec, and a final extension at 72°C for 7 min were performed.

An aliquot of 5 μl of the product of this nested PCR were electrophoresed in 2% agarose, stained with SYBR safe® (Invitrogen, CA), and visualized under UV light for the detection of a band of 550 bp. The remaining product was shipped to Macrogen (Macrogen Inc. Seoul, Korea) to be sequenced using the ED31 and ED33 primers (forward and reverse sequences, respectively).

The contigs were assembled using the Seqman™ II software version 5.01 (DNAStar Inc., Madison, WI) and the sequences obtained, including the V3 loop portion of gp120, served to predict the viral tropism using both the clonal and clinical (CCR5 Δ32 genotype, viral load, CD4+ T cell percentage and count, and CD8+ T cell count) approximations, by means of the geno2pheno (G2P) prediction algorithm (http://coreceptor.bioinf.mpi-inf.mpg.de/index.php). The reported false positive rate (FPR) was used as quantitative output and set to 20% [38].

### Data analysis

The overall activity of lovastatin vs. placebo in HIV-infected individuals was established by intention to-treat analysis. The frequency of positive cells and the mean fluorescence intensity of CXCR4 and CCR5 on CD4+ T lymphocytes at 0, 6 and 12 months after the intervention were compared by Generalized Estimating Equations (GEE) for repeated measurements, and informed as estimated average changes (EAC) with 95% Confidence Interval (CI). The frequency of positive cells and the mean fluorescence intensity of CXCR4 and CCR5 on CD4+ T lymphocytes from HIV negative controls at days 0, 7, 30 and 45 were compared by the one way Anova test for paired samples. The mean fluorescence intensity of CXCR4 and CCR5 on CD4+ T lymphocytes from asymptomatic HIV-1 infected patients vs. negative controls was compared by the Student´s t-test. The transcriptional levels of HIV co-receptors and their ligands during 45 days of oral administration of statins were compared by the one way Anova test for paired samples. The Fisher exact test was used to compare the frequency of X4 strains after one year of treatment in placebo- vs. lovastatin-treated groups. In all the comparisons, a value of p < 0.05 was considered statistically significant.

## Results

### Lovastatin administration does not induce a shift in HIV tropism

To determine the in vivo effect of lovastatin on the shift in viral tropism in HIV-1 infected subjects, a total of 91 DNA samples corresponding to month 0, and of 72 DNA samples corresponding to month 12 after daily administration of lovastatin, were amplified and sequenced. However, only 61 samples were paired (from the same patients at months 0 and 12) due to negative Env PCR amplification or low quality of the sequence obtained; 52.5% vs. 47.5% of the samples were from lovastatin and placebo patients, respectively. As shown in Table 2, using the clinical model of analysis, at baseline level, eight patients in the lovastatin treated group (18,1%) and eight (17,0%) in the placebo group were found with X4 X4/R5 circulating virus, and all were predicted to belong to the HIV-1 subtype B. A total shift in viral tropism of 15.6% in lovastatin-treated patients, and 20.7% in placebo patients was observed. The tropism shift from R5 to X4/X4R5 strains was 9.4% and 13.8% in lovastatin and placebo treated patients, respectively (p = 0.6988). Similar results were obtained using the clonal model of analysis (Table 2). These results suggest that, in this cohort of patients, the changes in use of the HIV-1 co-receptor were due to the natural course of infection and did not depend on the statin treatment.

**Table 2:** Prediction of co-receptor usage shift in proviral sequences from mononuclear cells

	**Total sequences obtained**	**Lovastatin**	**Placebo**	**P value (Fisher’s exact test)**
**Baseline**	91	44/91	47/9	NA
**(Number X4 X4/R5 strains)**		(8)	(8)	
**12 months post treatment**	72	38/72	34/72	NA
**Number of paired sequences**	61	32/61 (52.5%)	29/61 (47.5%)	NA
**Clinical model**	Total tropism shift	5/32 (15.6%)	6/29 (20.7%)	0.7426
	R5 shift to X4 or X4R5	3/32 (9.4%)	4/29 (13.8%)	0.6988
**Clonal model**	Total tropism shift	8/32 (25.0%)	5/29 (17.2%)	0.5411
	R5 shift to X4 or X4R5	4/32 (12.5%)	4/29 (13.8%)	1.0

The geno2pheno (G2P) algorithm was used at the beginning of the study and 12 months post treatment with lovastatin (40 mg/day) or placebo in asymptomatic HIV-1-infected patients. Isolation of peripheral blood mononuclear cells and proviral DNA preparation were as described in Methods. The G2P prediction algorithm is available at http://coreceptor.bioinf.mpi-inf.mpg.de/index.php. NA, No applicable statistical analysis. At the baseline were found 8 individuals with X4 or X4R5 circulating strains in lovastatin treated group, and 8 individuals at placebo treated group.

### Long-term use of statins does not affect the expression of HIV co-receptors on CD4+ T cells

To define in vivo if the long-term administration of lovastatin in HIV-infected patients reproduced a similar regulatory effect on the expression of CCR5 as previously shown in vitro [25], a cohort of chronically HIV-1 infected patients enrolled in the LIVE clinical trial [35] was examined. As shown in Table 3, one year administration of lovastatin had no significant effect on the percentage of CD4+ T cells expressing CXCR4 or CCR5 (EAC = 1.1%, 95% CI = −6.3 to 8.5; and EAC = 2.8%, 95% CI = −4.12 to 9.81, respectively). When the expression level of CXCR4 and CCR5 was measured as the mean fluorescence intensity (MFI) in the same population of CD4 + T cells, the results were similar (EAC = −39.2, 95% CI = −91.1 to 13.5 and EAC = −5.7, 95% CI = −23.9 to 12.4, respectively). The same analysis performed in the population of CD3-/CD4+ monocytes revealed no significant changes in the expression level of CXCR4 and CCR5 (Table 4).

**Table 3:** CXCR4 and CCR5 expression in CD4+ T lymphocytes

**Parameter**	**Time of treatment**	**Placebo group (n = 43)**	**Lovastatin group (n = 41)**
		**(95% CI)**	**(95% CI)**
**% CD4+/CXCR4+ T cells**	Baseline	68.5 (48.2 - 90.2)	58.5 (32.0 - 87.1)
	6 months post	60.0 (41.0 - 82.0)	66.4 (43.2 - 90.0)
	12 months post	58.5 (33.4 - 87.5)	66.0 (40.1 - 87.0)
	* EAC by treatment	**1.1% (−6.3 to 8.5)**	
**MFI CXCR4 in CD4+/CXCR4+ T cells**	Baseline	157.4 (63.6 - 221.0)	118.7 (76.0 - 176.4)
	6 months post	132.9 (44.4 - 195.6)	117.0 (72.1 - 200.7)
	12 months post	132.1 (35.3 - 231.4)	116.1 (39.3 - 184.6)
	**EAC by treatment**	**−39.2 (−91.1 to 13.4)**	
**% CD4+/CCR5+ T cells**	Baseline	17.8 (11.6 - 34.7)	23.6 (12.0 - 64.7)
	6 months post	19.2 (10.5 - 28.3)	26.0 (13.9 - 57.3)
	12 months post	21.5 (9.8 - 60.0)	22.5 (10.5 - 46.0)
	**EAC by treatment**	**2.84% (−4.1 to 9.9)**	
**MFI CCR5 in CD4+/CCR5+ T cells**	Baseline	67.9 (50.0 - 94.9)	71.6 (54.1 - 90.6)
	6 months post	63.0 (42.7 - 99.0)	66.2 (44.0 - 98.7)
	12 months post	58.4 (33.5 - 107.3)	83.5 (42.1 - 125.5)
	**EAC by treatment**	**−5.7 (−23.9 to 12.4)**	

CXCR4 and CCR5 expression levels were measured by flow cytometry at 0 (baseline) 6 and 12 months post treatment with lovastatin (40 mg/day) or placebo in asymptomatic HIV-1-infected patients. * EAC, Estimated average changes with (95% CI) using a GEE model as defined in Data Analysis (see Methods); MFI, Mean fluorescence intensity (in relative units).

**Table 4:** Expression of CXCR4 and CCR5 in CD3-/CD4+ monocytes

**Parameter**	**Time of treatment**	**Placebo group (n = 43) (95% CI)**	**Lovastatin group (n = 41) (95% CI)**
**% CD3-/CD4+/CXCR4+ monocytes**	Baseline	12.0 (3.0 - 23.0)	13.0 (4.0 - 21.0)
	6 months post	14.0 (8.0 - 24.0)	16.5 (5.0 - 29.0)
	12 months post	19.0 (10.5 - 37.5)	24.0 (10.0 - 58.0)
	**EAC by treatment**	**−2.2% (−7.2 to 2.8)**	
**MFI CXCR4 in CD3-/CD4+/CXCR4+ monocytes**	Baseline	196.8 (164.1 - 270.6)	192.2 (172.4 - 251.1)
	6 months post	179.7 (28.6 - 236.8)	189.2 (127.1 - 304.9)
	12 months post	149.6 (25.9 - 247.8)	86.2 (26.8 - 184.8)
	**EAC by treatment**	**8.7 units (−25.3 to 42.6)**	
**% CD3-/CD4+/CCR5+ monocytes**	Baseline	2.5 (1.0 - 6.0)	2.0 (1.0 - 8.0)
	6 months post	5.9 (1.3 - 16.5)	6.0 (2.3 - 12.2)
	12 months post	6.0 (3.0 - 35.0)	12.0 (6.0 - 33.0)
	**EAC by treatment**	**−0.99% (−5.1 to 3.1)**	
**MFI CCR5 in% CD3-/CD4+/CCR5+ monocytes**	Baseline	148.2 (131.7 - 155.9)	146.9 (127.9 - 167.6)
	6 months post	143.9 (38.1 - 168.2)	136.1 (48.9 - 171.0)
	12 months post	104.4 (21.0 - 169.6)	133.9 (33.4 - 164.6)
	**EAC by treatment**	**11.4 units (−28.0 to 50.9)**	

CXCR4 and CCR5 expression levels were measured by flow cytometry at 0 (baseline) 6 and 12 months post treatment with lovastatin (40 mg/day) or placebo in asymptomatic HIV-1 infected patients. * EAC, Estimated average changes with (95% CI) using a GEE model as defined in Data Analysis (see Methods); MFI, Mean fluorescence intensity (in relative units).

**Figure 1:**
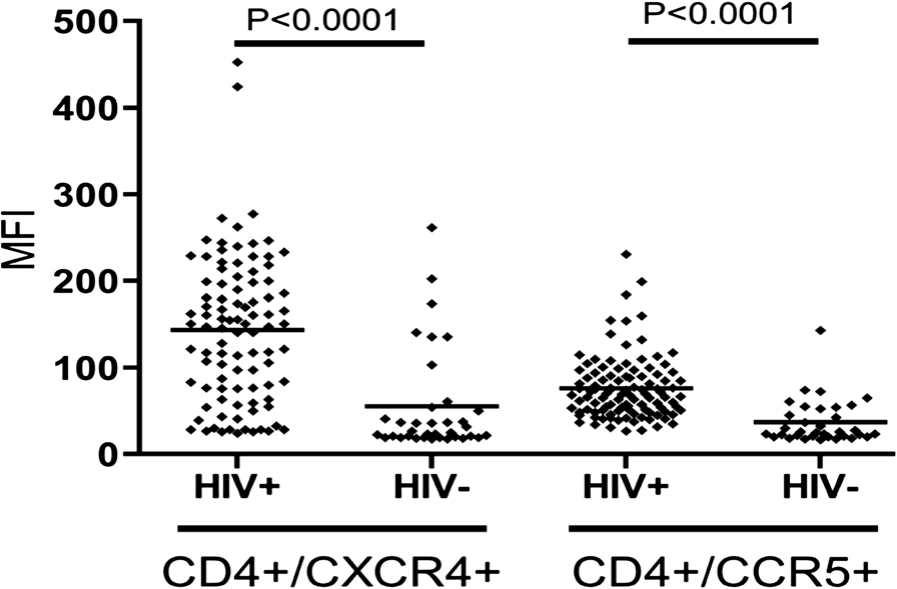
**Expression levels of CXCR4 and CCR5 in CD4+ T lymphocytes. A**: The mean fluorescence intensity (MFI) of CXCR4 and CCR5 was evaluated in CD4+/CXCR4+ and CD4+/CCR5+ T lymphocytes respectively, by flow cytometry in peripheral blood samples of 84 asymptomatic HIV-1-infected patients (HIV+) and 35 HIV seronegative volunteers (HIV-). Statistical comparison was made using the Student’s t-test.

Since the surface expression of CXCR4 and CCR5 could be modulated by the immune activation observed in HIV-1-infected patients, masking the effect of the long-term administration of oral lovastatin on the expression of these molecules, the baseline level of CXCR4 and CCR5 expression was evaluated and compared in HIV-1-infected patients and HIV negative controls. Indeed, as shown in Figure 1, the MFI for CXCR4 and CCR5 was significantly higher in CD4+ T cells of HIV-1-infected patients when compared to HIV negative individuals (median MFI CXCR4: 143 +/− 54 vs. 55 +/− 9 relative units, respectively, p < 0.0001; MFI CCR5: 76 +/− 36 vs. 37 +/− 26 units, respectively, p < 0.0001).

### Effect of statins on HIV co-receptor expression in HIV negative control subjects

Due to the differential expression of CXCR4 and CCR5 on resting CD4+ T cells of HIV-infected patients and HIV negative adults, we explored the effect of daily oral statins on the regulation of CXCR4 and CCR5 expression in HIV negative adults. At the different times of observation of statin oral intake, there were not statistically significant differences in the frequency and MFI expression level of CXCR4 by blood CD4+ T cells, when individuals receiving lovastatin or atorvastatin vs. controls were compared (Table 5). Regarding the expression of CCR5, only the administration of atorvastatin at 45 days of intake was associated with a significant increase in the frequency of circulating CD4+ T cells expressing CCR5 (median day 7 = 11.1%; day 45 = 71.4% ANOVA p < 0.05); there were no differences at other points of evaluation, in the CCR5 MFI level on CD4+ T cells, at different times and with the different treatments (ANOVA p ≥ 0.05) (Table 5).

**Table 5:** Follow-up of CXCR4 and CCR5 expression in CD4+ T lymphocytes

**CD4+ T lymphocytes**	**Median (interquartile range)**				**Comparison by Anova**
	**Day 0**	**Day 7**	**Day 30**	**Day 45**	
**% CXCR4+ cells**					
Controls without statins (n = 7)	56.2 (22.1 - 66.9)	45.2 (22.9 - 69.5)	68.4 (55.5 - 82.5)	69.2 (61.2 - 97.3)	NS
Lovastatin (n = 10)	54.5 (34.3 - 65.5)	30.9 (6.3 - 43.4)	66.1 (31.0 - 76.2)	63.8 (26.6 - 77.9)	NS
Atorvastatin (n = 9)	45.6 (35.6 - 64.4)	37.1 (24.8 - 44.6)	67.9 (51.0 - 75.6)	18.6 (7.1 - 66.9)	NS
**MFI CXCR4 in CXCR4+ cells**					
Controls without statins (n = 7)	19.9 (6.6 - 29.1)	15.8 (6.6 - 34.8)	25.1 (19.8 - 43.9)	21.2 (16.7 - 29.3)	NS
Lovastatin (n = 10)	16.5 (10.1 - 23.1)	8.9 (3.7 - 11.2)	16.3 (8.9 - 26.6)	20.4 (7.2 - 39.0)	NS
Atorvastatin (n = 9)	14.1 (10.8 - 27.3)	11.7 (7.8 - 13.2)	23.1 (15.1 - 29.3)	11.8 (4.3 - 33.8)	NS
**% CCR5+ cells**					
Controls without statins (n = 7)	17.4 (10.9 - 26.9)	28.2 (7.9 - 52.75)	35.0 (26.8 - 56.7)	21.6 (17.6 - 25.9)	NS
Lovastatin (n = 10)	13.3 (6.1 - 19.7)	9.4 (6.5 - 14.1)	14.1 (5.1 - 21.3)	18.1 (9.5 - 54.9)	NS
Atorvastatin (n = 9)	10.4 (7.6 - 18.7)	11.1 (7.1 - 12.0) £	13.5 (9.3 - 18.4)	71.4 (17.1 - 90.1) £	£ p < 0.05
**MFI CCR5 in CCR5+ cells**					
Controls without statins (n = 7)	8.8 (5.8 - 10.6)	13.2 (11.1 - 19.4)	11.1 (9.6 - 17.1)	25.1 (13.1 - 29.1)	NS
Lovastatin (n = 10)	23.0 (18.9 - 29.4)	21.9 (20.1 - 24.6)	29.4 (19.8 - 33.4)	24.1 (18.3 - 33.0)	NS
Atorvastatin (n = 9)	25.9 (20.4 - 50.2)	21.9 (20.0 - 25.4)	36.0 (27.0 - 42.4)	25.1 (23.4 - 39.6)	NS

HIV seronegative volunteers who received or not lovastatin (40 mg/day) or atorvastatin (20 mg/day) during 45 days, were evaluated at 0, 7, 30 and 45 days of treatment by flow cytometry. NS, No statistically significant difference. £: Statistical difference obtained comparing day 7 and day 45 of treatment.

In circulating monocytes, the administration of lovastatin for 45 days was not associated with significant modifications in the expression level of CXCR4 or CCR5 at the different times of evaluation (Table 6). In contrast, the daily oral administration of atorvastatin was associated with a significant increase in both molecules on peripheral blood monocytes, at days 30 and 45 of treatment when compared to the values at day 7 (median of cells expressing CXCR4, day 7 = 31.4%; day 30 = 72.3%; day 45 = 69.3%; ANOVA p < 0.05; MFI of CXCR4 day 7 = 8.6; day 30 = 25.0; day 45 = 22.4; ANOVA p < 0.05). Similar significant differences were observed for the percentage of CCR5+ monocytes and their CCR5 MFI, but only at day 45 of atorvastatin treatment (median of cells expressing CCR5, day 7 = 14.9%; day 45 = 76.1%; ANOVA p < 0.05; MFI of CCR5 day 7 = 6.2; day 45 = 26.8; ANOVA p < 0.05) (Table 6).

**Table 6:** Follow-up of CXCR4 and CCR5 expression in CD3-/CD4+ monocytes

	**Median (Interquartile range)**				**Comparison by Anova**
**CD3-/CD4+ monocytes**	**Day 0**	**Day 7**	**Day 30**	**Day 45**	
**% CXCR4+**					
Controls without statins (n = 7)	81.1 (61.0 - 83.5)	65.7 (41.9 - 85.4)	89.6 (55.6 - 93.0)	87.2 (60.7 - 91.2)	NS
Lovastatin (n = 10)	48.6 (43.7 - 76.3)	34.8 (16.1 - 50.2)	69.7 (51.5 - 81.6)	57.7 (37.2 - 88.9)	NS
Atorvastatin (n = 9)	40.2 (29.7 - 51.5)	31.4 (25.3 - 41.3) £. †	72.3 (53.6 - 82.4) £	69.3 (36.3 - 89.3) †	£ p < 0.05. † p < 0.05
**MFI CXCR4 in CXCR4+ cells**					
Controls without statins (n = 7)	34.6 (17.1 - 37.7)	22.9 (10.6 - 58.4)	40.1 (16.6 - 58.4)	29.9 (13.7 - 33.5)	NS
Lovastatin (n = 10)	16.3 (12.1 - 26.9)	9.2 (5.9 - 12.3)	19.5 (12.8 - 30.3)	13.4 (9.6 - 64.9)	NS
Atorvastatin (n = 9)	10.6 (10.1 - 14.5)	8.6 (7.6 - 10.3) £. †	25.0 (13.5 - 28.2) £	22.4 (9.7 - 57.9) †	£ p < 0.05. † p < 0.05
**% CCR5+**					
Controls without statins (n = 7)	56.8 (32.2 - 64.5)	52.9 (15.7 - 70.3)	60.9 (32.1 - 72.3)	79.6 (27.8 - 80.0)	NS
Lovastatin (n = 10)	27.3 (21.6 - 41.5)	22.7 (7.8 - 26.9)	49.2 (24.0 - 61.8)	50.5 (18.9 - 73.7)	NS
Atorvastatin (n = 9)	23.6 (17.0 - 55.7)	14.9 (8.5 - 21.3) †	55.7 (25.8 - 57.2)	76.1 (27.3 - 78.3) †	† p < 0.05
**MFI CCR5 in CCR5+ cells**					
Controls without statins (n = 7)	15.9 (9.0 - 19.4)	16.4 (6.0 - 25.2)	16.1 (10.2 - 29.0)	18.3 (8.0 - 20.7)	NS
Lovastatin (n = 10)	8.6 (7.0 - 13.3)	7.1 (4.7 - 8.3)	12.7 (7.5 - 18.5)	11.8 (6.4 - 27.2)	NS
Atorvastatin (n = 9)	7.1 (6.3 - 19.8)	6.2 (4.7 - 7.5) †	15.3 (8.0 - 16.1)	26.0 (8.0 - 29.5) †	† p < 0.05

HIV seronegative volunteers who received or not lovastatin (40 mg/day) or atorvastatin (20 mg/day) during 45 days, were evaluated at 0, 7, 30 and 45 days of treatment by flow cytometry. NS, No statistically significant difference. †: Statistical analysis performed comparing day 7 and day 45 of treatment; £: Statistical difference obtained comparing day 7 and day 30 of treatment.

### Effect of statins on the transcription level of HIV co-receptors and their ligands in HIV negative controls

**Figure 2:**
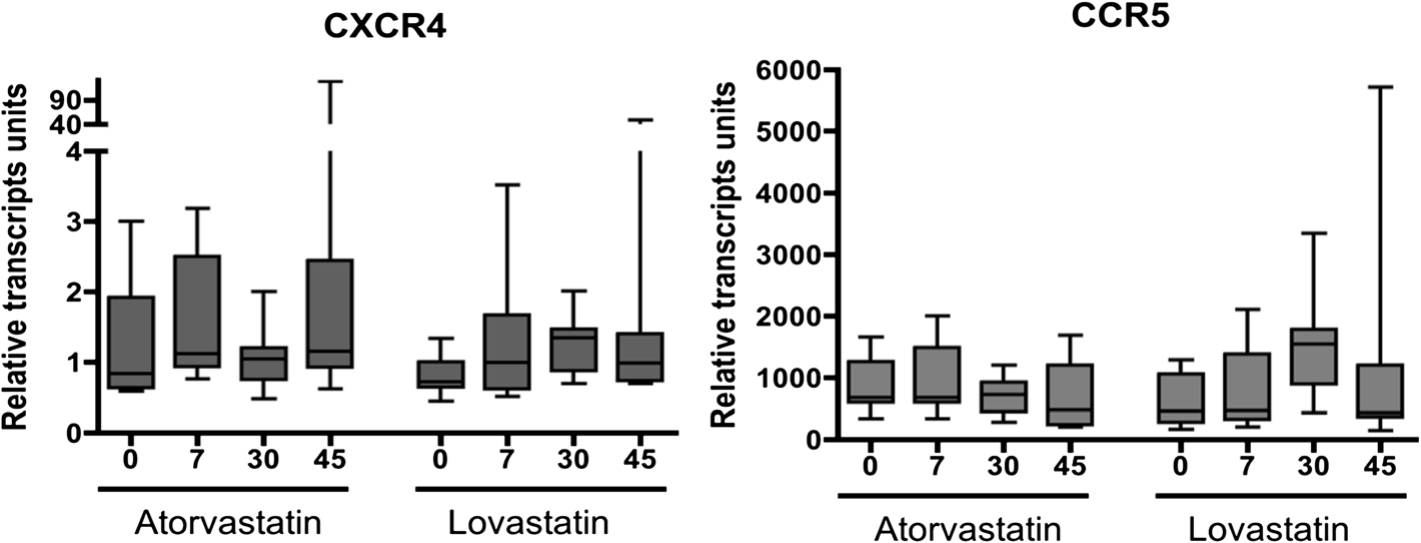
**Transcription levels of CXCR4 and CCR5 genes during lovastatin or atorvastatin treatment.** Transcription levels of CXCR4 and CCR5 in purified CD4+ T cells of adult HIV negative volunteers, during 45 days of oral administration of atorvastatin (20 mg/day; n = 9) or lovastatin (40 mg/day; n = 10); the measurements were performed by real time PCR at baseline and on days 7, 30 and 45. Statistical comparisons were performed using the One Way Anova test for paired samples.

**Figure 3:**
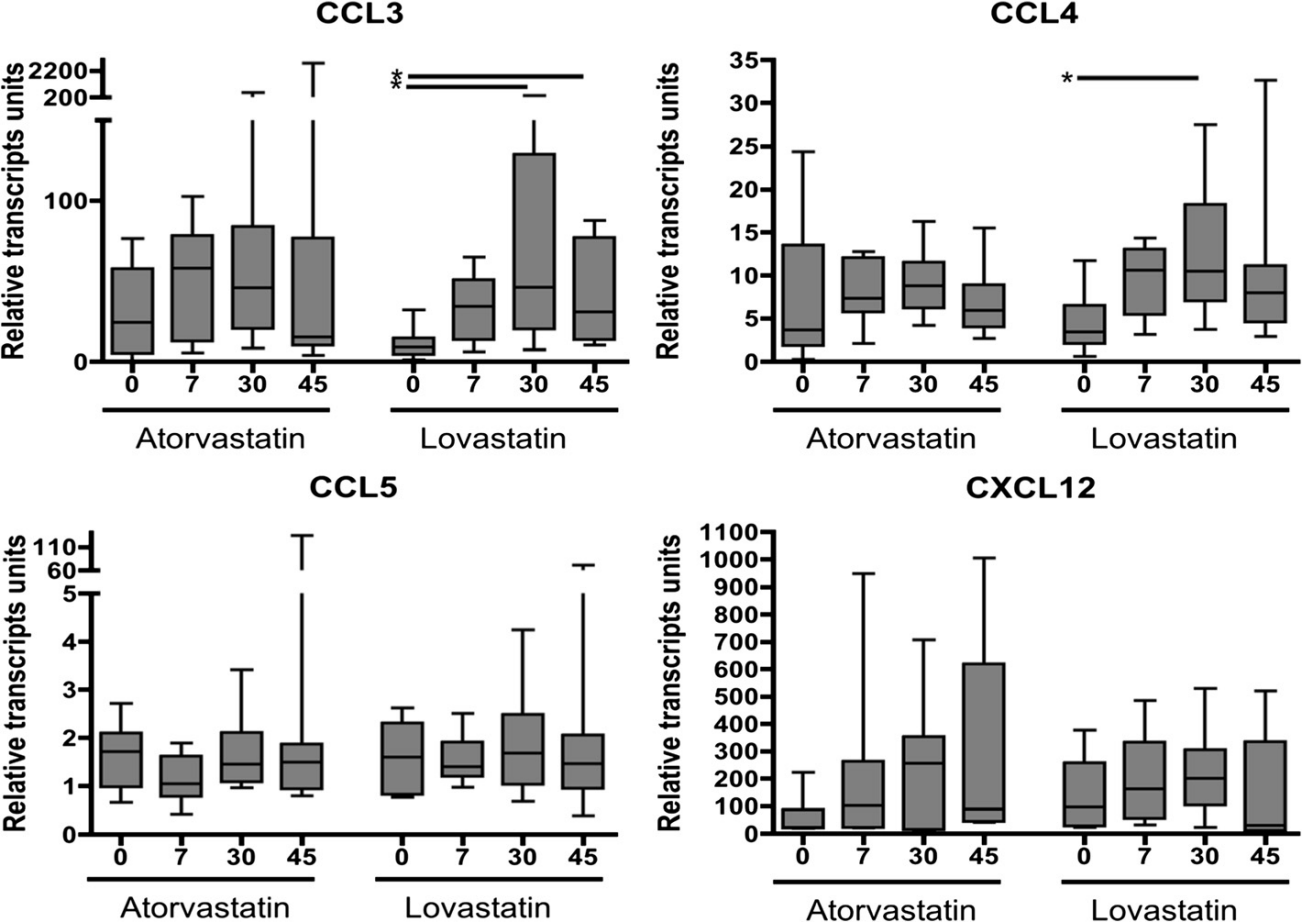
**Transcription levels of CCL3, CCL4, CCL5 and CXCL12 genes during lovastatin or atorvastatin treatment.** Transcription levels of CCL3, CCL4, CCL5 and CXCL12 in purified CD4+ T cells of adult HIV negative volunteers, during 45 days of oral administration of atorvastatin (20 mg/day; n = 9) or lovastatin (40 mg/day; n = 10); the measurements were performed by real time PCR at baseline and on days 7, 30 and 45. Statistical comparisons were performed using the One Way Anova test for paired samples. (*): p value <0.05, considered significant.

Daily administration of lovastatin or atorvastatin was not associated with significant differences at the transcription level of the CXCR4 and CCR5 co-receptors at the different times of sampling (ANOVA p ≥ 0.05) (Figure 2). Similarly, the transcriptional activity of CCL5 and CXCL12 in CD4+ T cells was not significantly modified by the daily administration of lovastatin or atorvastatin (ANOVA p ≥ 0.05) (Figure 3, lower panel). In contrast, the administration of lovastatin was associated with a significant increase in the transcription of CCL3 from 0 to day 30 and 45 of treatment (median relative units of transcription = 8.85, 46.71 and 30.78 respectively, ANOVA p < 0.05), and in the transcription level of CCL4 at day 30 (median relative units of transcription day 0 = 3.4; day 30 = 10.6, ANOVA p < 0.05 (Figure 3, upper panel); however, the administration of atorvastatin was not associated with significant variations in the transcriptional activity of CCL3 and CCL4 (Figure 3, upper panel).

## Discussion

In this investigation, evaluating a representative cohort of chronic asymptomatic HIV-1-infected patients without requirements to receive antiretroviral therapy, we demonstrated that a daily oral administration of lovastatin during one year did not result in significant modulation of CCR5 and CXCR4 expression on CD4+ T cells or CD4+ monocytes. Also, in HIV negative individuals, the daily administration of two different statins for 45 days did not lead to a significant regulation of these molecules. These findings suggest that long-term administration of statins, at doses commonly used in clinical practice, does not have a significant effect on modulating the expression of HIV-1 co-receptors or their ligands (except for CCL3 and CCL4).

Previous results support the immunomodulatory effects of statins in different inflammatory states [27,39-46]. Similarly, the in vitro modulation of the expression of CCR5 and RANTES in CD4+ T lymphocytes by statins, as well as their inhibition of infection by R5 strains of HIV-1, was previously demonstrated [25]. These findings highlighted the importance of verifying whether the administration of statins modulated in vivo the expression of such a receptor and its ligands, and if this treatment could lead to the selection of X4 tropic strains of HIV-1 in infected individuals. Several explanations might account for the apparently contradictory data obtained in the in vitro studies and our in vivo studies regarding the actions of statins. The incubation of CD4+ T cells with statins in well plates allows the direct interaction of these compounds with their cellular targets (HMG-CoA reductase), favouring the molecular effects expected of statins. However, this might not be the case in vivo; considering that HIV replication and immune activation are active on secondary lymphoid organs (lymphoid nodes, mucosal lymphoid tissues), several pharmacokinetic limitations could hamper the effective biological activity of statins. In particular, if the bioavailability of statins in lymphoid tissues is deficient, then the immunomodulatory activities of these compounds will be limited. Thus, statins with better pharmacokinetic profiles would be more suitable to achieve the effects observed in vitro, where direct administration of statins to cells in culture overcomes the potential limitations derived from oral administration. It is also possible that long-term administration of higher doses of statins might be associated with better immunomodulatory effects, but the risks of adverse events and intolerance also increase, questioning the beneficial balance of such doses. Due that several factors could impact the outcome, including drug dose, potency, treatment-time, further investigations are needed in order to carefully demonstrate that statins do not affect the expression of coreceptors and their ligands in HIV infected or uninfected individuals.

The steady state expression of HIV-1 co-receptor molecules has been previously defined in HIV negative individuals, indicating that peripheral blood CD4+ naive T cells (CD45RA+) express high levels of CXCR4, but most of them are negative for CCR5; in contrast, most memory CD4+ T cells express CCR5, becoming the main target cells for efficient HIV-1 replication [47]. Also, in memory T cells, the expression of CCR5 may be modulated by cell activation [17,48-50]. Other observations support the fact that the level of CCR5 expression is associated with the infectious capacity of R5 strains of HIV [51] and give us an important evidence that if we regulate the expression of CCR5 it might act as a target for HIV infection prevention and control of replication. Additionally, HIV gp120 can acts in lymphocytes as “viral chemokines” interacting with CCR5 or CXCR4, and activating downstream signal proteins, induction of ion channel currents and Ca^2+^ flux, quantitatively different to that induced by chemokines. These differences in gp120 and chemokine-elicited Ca^2+^ flux can result in a Long Terminal Repeat (LTR) activation, due to a possible differential transcription factor activation [52]. The importance of the density of CD4 and of HIV CCR5 co-receptor expression for efficient infection has been also investigated in other cells different from human CD4+ T cells. In CD4+ HeLa cells expressing minimal concentrations of CCR5 on their surface, the infection by R5 strains of HIV occurred more efficiently during CCR5 expression concomitantly with a high level of CD4 expression, suggesting that a minimal number of CCR5 molecules are required when cell surface levels of CD4 reach a sufficient density [53]. Along the same line, epidemiological results associated the Δ32 CCR5 homozygous genotype with protection against the acquisition of HIV-1 [54-57], indicating that modulating CCR5 expression or function in HIV-infected individuals could help control progression of HIV infection, as occurs with maraviroc treatment; in despite of this, some Δ32 CCR5 homozygous individuals have become infected with HIV-1, indicating that this genotype may not be fully protective [58,59]. In our study, when compared with HIV negative baseline values a significant increase in the level of CCR5 expression on peripheral blood CD4+ T cells from the HIV-1 infected individuals was observed, which is in agreement with the uncontrolled chronic immune activation characterizing this infection. Furthermore, expression of CXCR4 on these cells was also significantly increased in infected individuals. Taken together, these facts suggest that generation of target cells with higher expression of co-receptors requires niches for persistent viral replication, and could be another pathological event associated with chronic immune activation during HIV infection.

Increased CXCR4 expression in HIV-infected patients is a remarkable finding, since enhanced expression of CXCR4 has been associated with more risk of metastasis in different types of cancers [60-64]; in fact, increased severity of oncogenic processes in chronically HIV-infected individuals has been previously documented [65-71]. These findings highlight the importance of investigating the association between chronic immune activation, enhanced expression of CXCR4 by different cells and cancer progression, and metastasis during HIV infection.

Regarding the expression of the CXCR4 and CCR5 ligands by purified CD4+ T cells of HIV negative adults, we observed an increase in CCL3 and CCL4 transcripts during lovastatin but not during atorvastatin treatment. The increase in both transcripts might correspond to the fact that the CCL3 and CCL4 genes are complexed in a very close genomic region, and their expression is determined by the number of gene copies [72]. It is possible that differences in the chemical structure, potency and molecular activity of lovastatin (natural statin) vs. atorvastatin (synthetic statin) could be associated with the apparently distinct immunomodulatory activities of both statins.

Previous in vitro evidence indicated that statins were not efficient in inhibiting the infection of CD4+ T cells by X4 strains of HIV-1, in contrast to their capacity to hamper the infection by R5 strains [25]; these observations generated the hypothesis that the long-term administration of statins to HIV-1-infected patients could lead to the selection of X4 strains, contributing to accelerate the evolution of HIV infection to AIDS. The frequency of the shift of R5 strains to X4 strains was similar between placebo and patients treated with lovastatin for one year, suggesting that the shifts in co-receptor usage were due to the natural course of infection, rather than to a selective process induced by statins. This finding suggests that the therapeutic administration of statins in HIV-infected patients with lipid disorders could be safe in terms of selecting X4 strains.

Although the role of the CCR5 and CXCR4 co-receptors in HIV-1 infection is well established, little is known regarding the regulation of their expression. Understanding these processes could facilitate the development of new therapeutic interventions with a similar mechanism of action as maraviroc. The investigation of new compounds that block or modulate the expression and function of HIV co-receptors must continue, since they represent conserved host molecules rather than viral proteins susceptible to continued mutations, making them interesting targets for anti-viral therapies.

## Competing interests

EAH received a doctoral scholarship from COLCIENCIAS; the other authors declare that they have no competing interest.

## Authors’ Contributions

EA was the primary researcher, conceived the study, designed, participated in data collection and drafted manuscript, FJ assigned the randomization of patients and conducted statistical analysis, MR and CM assisted in data collection, follow-up the HIV positive patients and reviewed the initial and final drafts of the manuscript. All authors read and approved the final manuscript.
